# A Study on the Reliability Evaluation of a 3D Packaging Storage Module under Temperature Cycling Ultimate Stress Conditions

**DOI:** 10.3390/mi15040428

**Published:** 2024-03-23

**Authors:** Shuai Zhou, Kaixue Ma, Yugong Wu, Shizhao Wang, Nian Cai

**Affiliations:** 1 School of Microelectronics, Tianjin University, Tianjin 300072, China; zhoushuai@tju.edu.cn (S.Z.); wuyugong@tju.edu.cn (Y.W.); 2 China Electronic Product Reliability and Environmental Testing Research Institute, Guangzhou 511370, China; 3 School of Power and Mechanical Engineering, Wuhan University, Wuhan 430072, China; wangshizhao@whu.edu.cn; 4 School of Information Engineering, Guangdong University of Technology, Guangzhou 510006, China; cainian@gdut.edu.cn

**Keywords:** 3D packaging, temperature cycling, reliability enhancement test, ultimate stress

## Abstract

Based on the theory of reliability enhancement testing technology, this study used a variety of testing combinations and finite element simulations to analyze the stress–strain properties of 3D packaging storage modules and then evaluated its operating and destruction limits during temperature cycling tests (−65 °C~+150 °C) for the purpose of identifying the weak points and failure mechanisms affecting its reliability. As a result of temperature cycling ultimate stress, 3D packaging storage devices can suffer from thermal fatigue failure in the case of abrupt temperature changes. The cracks caused by the accumulation of plastic and creep strains can be considered the main factors. Crack formation is accelerated by the CTE difference between the epoxy resin and solder joints. Moreover, the finite element simulation results were essentially the same as the testing results, with a deviation occurring within 10%.

## 1. Introduction

During manufacturing and usage processes, 3D packaging storage is usually subjected to various forms of environmental stress, such as thermal, mechanical, and hygrothermal. According to the *Electronic Components Failure Analysis Manual* [1], it can be observed from the statistics that the largest proportion of failures occurring in electronic components is caused by thermal stress, accounting for approximately 55% of the total cases. In regard to the interlinked structure inside 3D packaging storage, in addition to the thermal expansion mismatch between adjacent materials, in the events of periodic high–low temperature alternations or an extreme temperature gradient, changes occur in a considerably easier manner in the internal stress and microstructure of a solder joint, so that the stress–strain pressure inside the solder joint is continuously accumulated, eventually resulting in cracks in or the thermal fatigue failure of the solder joint.

For the qualification tests of microelectronic devices, it is necessary to conduct a range of tests (e.g., physical, temperature, moisture, mechanical, and lifespan tests) in accordance with the procedures and conditions specified in the relevant general specifications, test standards, and product manuals to verify whether the device quality and reliability of products meet the corresponding requirements [2], which is also regraded as an important link in device development and finalization practices. Institutions, such as the National Aeronautics and Space Administration (NASA) and the European Space Agency (ESA), explicitly stipulate that device products that have passed the identification and testing stages should be prioritized in high-reliability applications [3,4]; however, many problems, such as “bad use” and “poor usability”, still exist in the devices used during the application process. The main reasons for these problems include the fact that that there is a discrepancy between the standardized test methods and the applications of the devices and technology, and that the device’s stress profile cannot meet the demands of all the device application states, especially in extreme environmental applications where the device also experiences various failures [5,6,7]. Therefore, it is urgent to evaluate the ultimate capacity of microelectronic devices and determine their application boundaries through a new quality reliability evaluation method to guarantee their long-term application reliability.

The reliability enhancement test (RET) constantly follows the principle of “exposing the defects of a device as much as possible without exceeding the destruct limit of the device” and loads stress in a stepwise or constant manner to obtain or evaluate the operating limit of the device, thereby providing evidence for its design improvement in complex and harsh environments [8,9]. The reliability enhancement testing technology used for evaluating the ultimate capacity of devices and improving their quality reliability has been widely adopted in many fields both at home and abroad, including aerospace, communications, medical treatment, and manufacturing, since the late 1980s and early 1990s [10,11]. However, only a few studies conducted on microelectronic devices exist in the literature. In addition, the studies performed on devices with complex packaging structures, such as 3D packaging, have not yet been publicly published. On the basis of the theory of reliability enhancement testing technology, this study, using a certain 3D packaging storage module as the research object, analyzes its failure modes and mechanisms under a temperature cycling test condition and then evaluates the ultimate stress level of temperature cycling through a variety of tests and a finite element analysis.

## 2. Reliability Enhancement Testing Technological Theory

Proposed by American researchers, G.K. Hobbs, K.A. GRAY, and L.W. Condra et al., the RET technological theory takes the highly accelerated stress test (HAST) and the highly accelerated stress test (HASS) as the theoretical bases of the RET technology [12].

**Figure 1:**
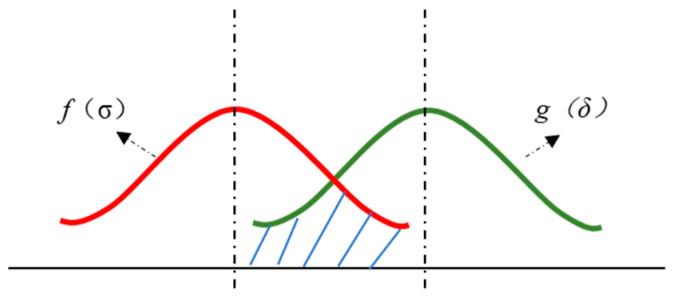
Probability density distribution curves of stress and strength.

As a provocation testing method, RET takes failure as the main research object, and quickly stimulates and eliminates potential defects to improve the product reliability by applying harsh environmental and working stress that exceeds the actual application conditions to the sample [13]. This is one of the biggest differences from standardized identification test techniques. The factors that lead to product failure can be summarized as stress and strength. Stress covers all factors that can result in product failure, while strength is the general name of all factors for the product to resist failure. If the stress borne by the product is smaller than its strength, the product will not fail, otherwise it will. Affected by factors such as working environment and load, both stress and strength are variables that obey specific random distribution patterns. *f*(*σ*) was assumed to be the probability density function of stress and *g*(*δ*) was denoted as the probability density function of strength. The probability densities of the two were plotted in the same coordinate system to obtain the probability density distribution of stress and the strength of a specific component (Figure 1). 

**Figure 2:**
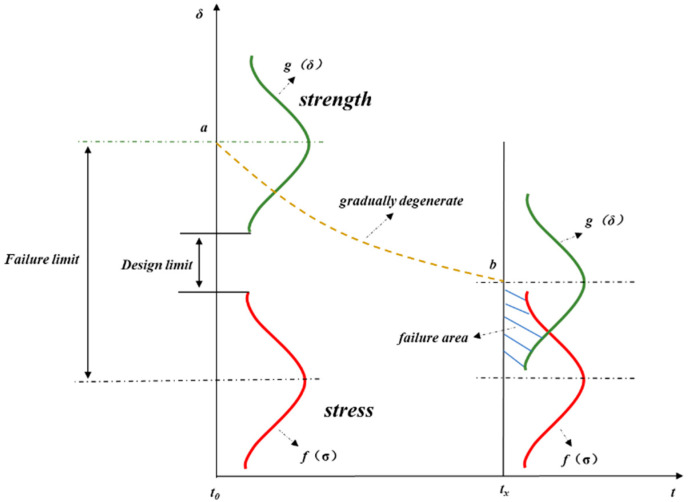
Change in reliability with working time.

In product design, a product’s nominal strength should be higher than its nominal stress. However, due to the discreteness of stress and strength values, their probability density curves may intersect in some areas, that is, stress and strength will interfere, as shown in the shaded part in Figure 1. In this interference area, the strength value may be smaller than the stress value, which indicates a risk of failure. Even though the stress and strength probability density curves of the product do not interfere at the initial stage, the product’s strength will gradually deteriorate after long-term use under mechanical shock, temperature cycles, and damp heat [14]. As shown in Figure 2, the average intensity declines from a at time *t*_0_ to *b* at time *t_x_*, and the safety margin *h* of conventional design decreases as well. Hence, with the passage of working time, the reliability of the product gradually decreases until it fails.

Figure 2 shows that when there is a high degree of dispersion between the strength and stress of a product, the interference area between the two will increase and the product’s failure rate will be increased, and vice versa. The product fails only when its stress surpasses its strength limit. According to the stress–strength probability density curve, the potential defects of the product can be stimulated at a faster rate by applying the strengthened stress beyond the designed strength limit of the product, and short-term exposure of the product’s weak links of quality reliability is much more efficient than the other traditional reliability tests, which is also the basic principle of RET.

**Figure 3:**
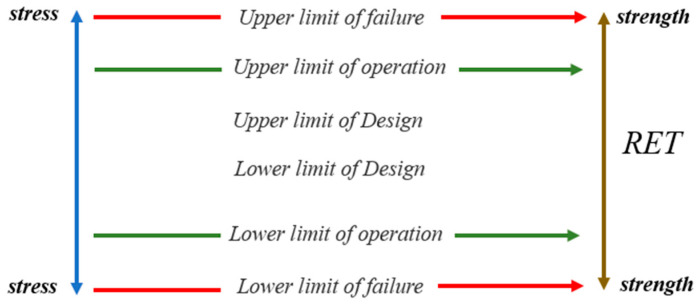
Different limiting ranges of stress of the product. Reliability enhancement testing simulation of the temperature cycle.

A product’s limiting ranges of stress can be divided into the following three types (Figure 3): (1) the technical specification limit is the absolute maximum rating value specified by the product manufacturer or designer; (2) the working limit, also known as the available limit, refers to the absolute maximum rating value that enables the product to work normally without irreversible failure within the range greater than the designed limit; (3) the failure limit represents the absolute maximum rating value for the product to experience functional failure or out-of-tolerance performance parameters. Adhering to the principle of “exposing product defects as much as possible within the product’s failure limit”, RET loads stress in a step-by-step or constant way, and then obtains/evaluates the working limit of the product, thereby providing a basis for improving the design of the product so as to make it applicable to complex and harsh environments.

### 2.1. Structural Parameters and Modeling of 3D Packaging Memory

**Figure 4:**
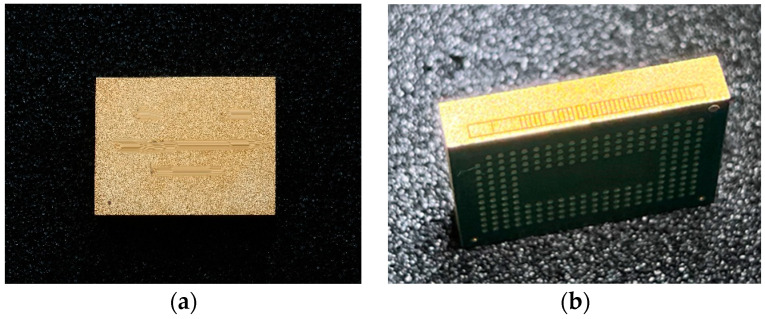
Typical appearance of a 3D packaging storage module: (**a**) front; (**b**) profile.

**Figure 5:**
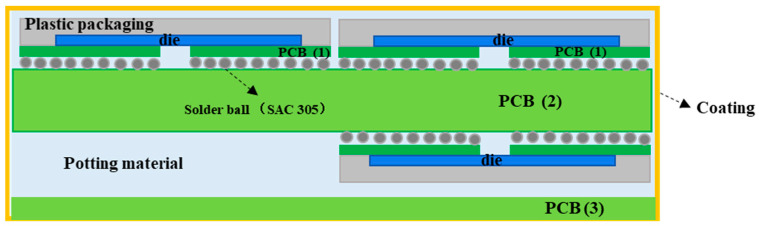
Internal structure and appearance.

The 3D packaging storage module selected in this study was stacked with five DDR3 SDRAM chips with a capacity of 256Mx16 bits. The entire module was divided into two PCB layers for stacked packaging, with a bottom board on the first layer and a function board on the second layer. Four chips were welded onto the front of the function layer, with one chip and resistor–capacitor on the back. Then, the lead wires of the chip and lead frame layers were connected in a 3D plastic packaging structure through gold-plated cables (nickel–copper–nickel–gold-coating structure) on the outer surface after being potted and cut. As a result, the DD3 SDRAM BGA storage module with a capacity of 256 M × 72 bit was determined. The structure features and morphology are presented in Figure 4 and Figure 5, and the structure dimensions are presented in Table 1.

**Figure 6:**
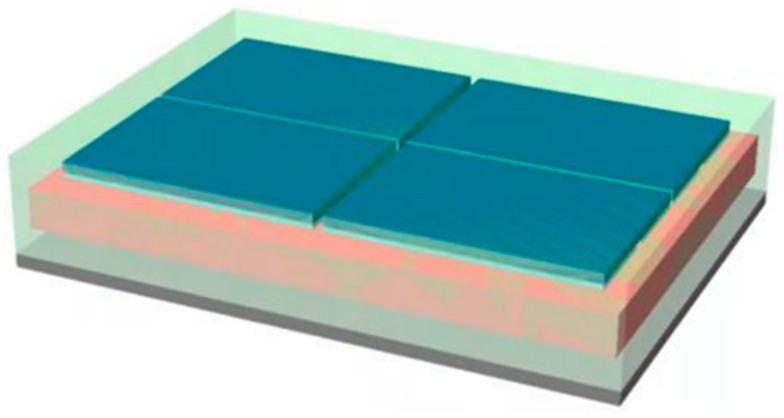
Three-dimensional structure and appearance of the storage module.

**Figure 7:**
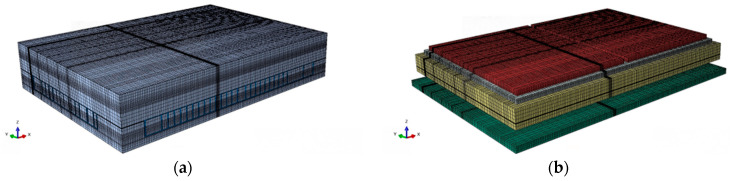
The entire model: (**a**) interconnected structure and resin sealing compounds; (**b**) interconnection structure.

**Figure 8:**
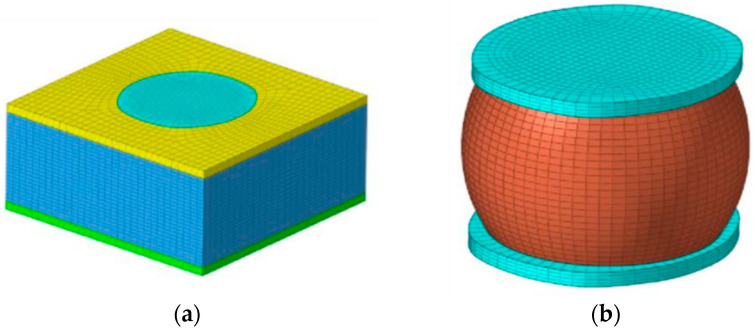
Sub-model: (**a**) solder joints and resin sealing compounds; (**b**) solder joints.

Based on the dimensions and relative positions of various components inside the storage module, a three-dimensional model was established using Abaqus simulation modeling software [15], as shown in Figure 6, Figure 7 and Figure 8. To appropriately simplify the model mesh and improve the convergence of the model, square solder joints were used instead of spherical solder joints to ensure the continuity between the solder joint and filling material meshes.

The module was mainly composed of a PCB, chip, substrate, plastic sealing compound, resin sealing compound, and external nickel plating. This model mainly included the thermophysical properties of the compounds, and it was assumed that the physical properties of each material could not vary with temperature. The constitutive model of Anand was used in the internal compound of solder balls endowed with the creep property, while the remaining compounds were set to be linear elastic. Moreover, the PCB compound was set to provide an orthotropic expansion, while the remaining compounds were isotropic in nature. The properties of various compounds are presented in Table 2.

### 2.2. Setting and Loading of Temperature Cycling Test Conditions

Different test conditions (temperature, load time, and switching time) present in a temperature cycling test can exert an influence on the failure mode and mechanism of a device. Hence, it is necessary to consider the storage temperature range (+150 °C~−65 °C) of microelectronic devices in the field of high-reliability applications, and maintain the same failure mechanism in qualification tests, so that the 1010 condition C of the temperature cycling test method, according to the standard MIL-STD-883 *Test Methods and Procedures for Microelectronic Devices*, can establish the temperature cycling curve.

**Figure 9:**
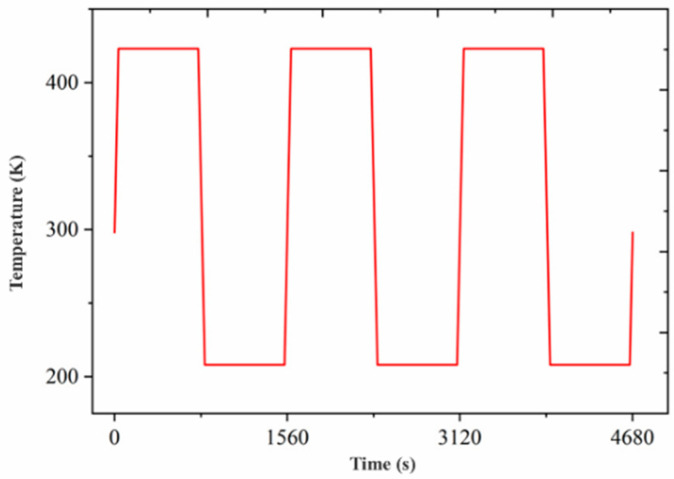
Temperature cycling load curves.

A temperature cyclic curve is a type of curve used to describe the change in temperature of an object over time during a temperature change. Typically, a temperature cycling curve is a curve plotted with time as the horizontal coordinate and temperature as the vertical coordinate. The drawing method of temperature cycling curve can be used to select different temperature change ranges and time intervals according to the actual needs in order to simulate the actual use conditions of the product or a specific environment. By analyzing the temperature cycling curve, the stress–strain and thermal stability of the product under the temperature change environment can be effectively evaluated and analyzed, which is of great significance and usefulness for the improvement of the design of the product and for improving the quality and reliability. It is widely used in various engineering fields. The temperature cycling load can be selected for simulation purposes, including the temperature range (−65 °C~+150 °C), the switching time of 1 min from hot to cold or from cold to hot temperatures, and the load time of 12 min at high or low temperatures. The relevant temperature cycling load curves are presented in Figure 9. For most of the metal materials tested, during the simulation, the increasing cycling number after 3–5 cycles resulted in a minor deviation in the stress distribution, below 1.5%. However, this required a significant increase in the computational cost. Therefore, three cycles were performed for the load application during this simulation process.

**Figure 10:**
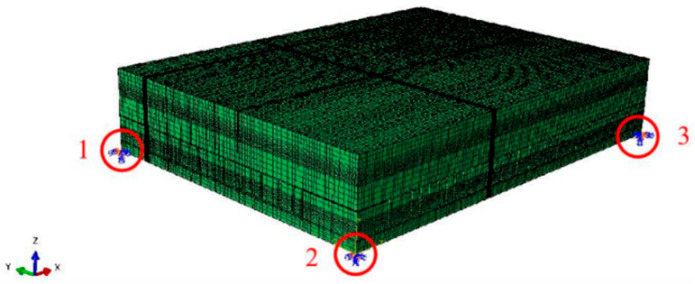
Boundary conditions of the entire model(Point 1, Point 2, Point 3).

The position of the entire model was constrained by fixing it at three points. U denotes the degree of freedom along the axial direction, U1, U2, and U3 denote the degree of freedom along the x-axis, y-axis, and z-axis, respectively; UR denotes the rotational degree of freedom around the axial direction; and UR1, UR2, and UR3 denote the rotational degree of freedom around the x-axis, y-axis, and z-axis, respectively. It can be observed in Figure 10 that by applying the boundary condition of U3 = UR1 = UR2 = UR2 = 0 to points 1, 2, and 3; U1 = UR2 = UR3 = 0 to points 1 and 2; and U2 = UR1 = UR3 = 0 to points 2 and 3, the module’s corners are clamped down, where two points are fixed to three of the device’s sides. The sub-model was constrained by strain loads based on the sub-model boundary conditions configured by the Abaqus software used. Moreover, the temperature load was directly applied to the entire model.

The quasi-static analysis (visco) provided by Abaqus software can be used to analyze time-dependent material response issues (for example, creep, swelling, viscoelasticity, and viscoplasticity). Based on the quasi-static analysis, the creep of the solder joint was calculated. According to the automatic incremental calculation, a total time of 4680 s was achieved in a time frame of three cycles, with alternative parameters being set as default values.

### 2.3. Analysis of the Simulation Results

#### 2.3.1. Analysis of the Temperature Cycle Model

**Figure 11:**
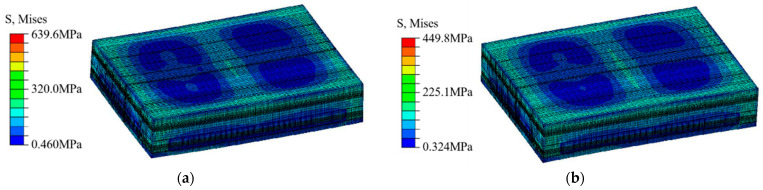
Von Mises stress nephograms: (**a**) stress value at +150 °C; (**b**) stress value at −65 °C.

The von Mises stress nephograms of the output model at the low temperature of −65 °C and high temperature of +150 °C indicate that the module has already experienced convex warping at a low temperature and concave warping at a high temperature. As a result of the thermal expansion coefficient of the chip being lower than that of the welding ball area, the stress–strain level in this area was relatively low, as shown in Figure 11.

**Figure 12:**
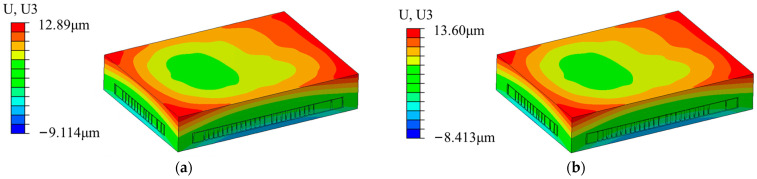
U3 deformation nephograms of the model following temperature cycling: (**a**) fixing the lower left corner of the bottom of the model; (**b**) fixing the upper right corner of the bottom of the model.

Figure 12a shows the U3 deformation nephogram at the high-temperature stage in the first cycle. It can be observed that the deformation that occurred in the area located at a considerable distance from the B-side chip is relatively small; therefore, the overall deformation of the module is asymmetric, possibly resulting from the asymmetric distribution of the B-side chips. Since the boundary condition of this model was constrained by fixing the lower left corner of the bottom of the model, it should be reset to constrain the upper right corner of the bottom section to avoid the influence of the constraint conditions; the U3 deformation nephogram is presented in Figure 12b. It can be observed that the model’s deformation gradient is almost consistent with the original model, with only slight numerical differences being evident, indicating that the abovementioned phenomenon is caused by the structure of the model itself.

**Figure 13:**
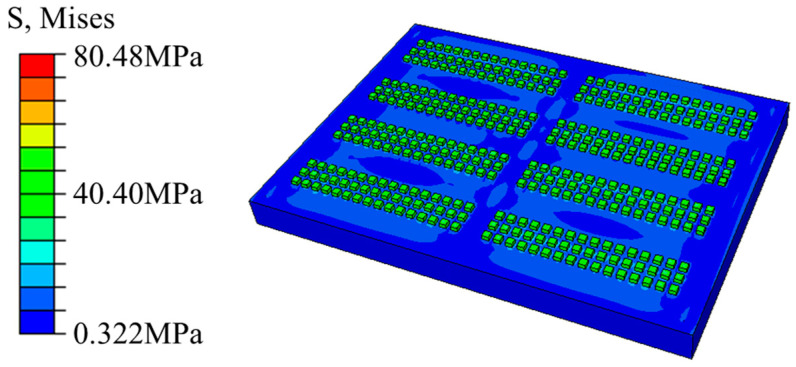
Stress nephogram of the internal structure of the model.

The internal von Mises stress nephogram of the model, following the temperature cycling stage, is presented in Figure 13. It can be observed that the stress that occurs after the temperature cycling stage is mainly concentrated in the solder joint area, with a maximum stress value of 80.48 MPa present at the junction between the solder joint and the intermediate PCB. When the von Mises stress is considered to be a damage criterion, the area is dangerous.

**Figure 14:**
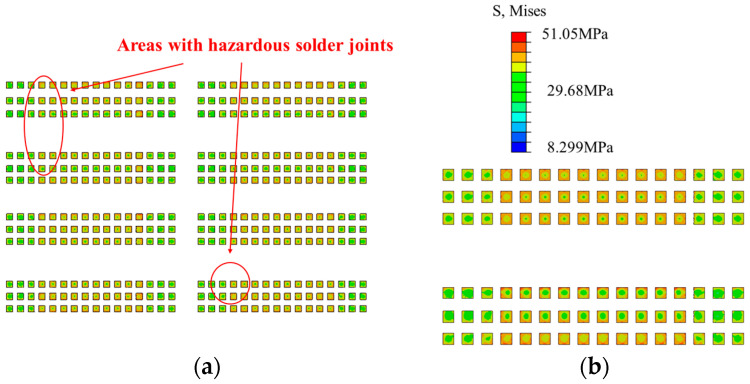
The von Mises stress values of the solder joints following temperature cycling and load cycling techniques: (**a**) A-side stress distribution; (**b**) B-side stress distribution.

The distribution of von Mises stresses at the A-side and B-side solder joints after temperature cycling is presented in Figure 14. From the stress nephogram, it can be observed that there is no significant difference in the stress distribution trend of the corresponding solder joints for each chip. The solder joints inside a single chip are divided into two parts, with the solder joints located below the chip representing chip-end solder joints, while the solder joints located below the plastic package layer represent package-end solder joints. It is obvious that the von Mises stress occurring at the chip-end solder joints is significantly higher than that at the package-end solder joints. Moreover, within a single chip, the stress on the contact surface between the solder joints and the chip shows a clear trend of a diffusion distribution along the center of the chip. The closer it is to the chip edge, the greater the solder joint stress. The reason for this phenomenon is that the minor deformation of the chip during the temperature cycling process occurs due to its relatively small thermal expansion coefficient, while the large thermal expansion coefficient of the device substrate below the chip results in its high deformation rate during the temperature cycling process. The deformation of the chip limits the deformation of the solder joints at the connection with the substrate, causing a significant stress on the contact surface of the solder joints. Additionally, since the chip size is approximately square, its thermal expansion coefficient is anisotropic, indicating that the closer it is to the chip edge, the greater the strain of the chip. Therefore, failure is most likely to occur at the diagonal solder joints. As shown in Figure 14, the marked area refers to the hazardous area of the chip solder joints located on the upper left side of the A-side module, while the hazardous solder joints of other chips also tend to be visible in the diagonal area.

**Figure 15:**
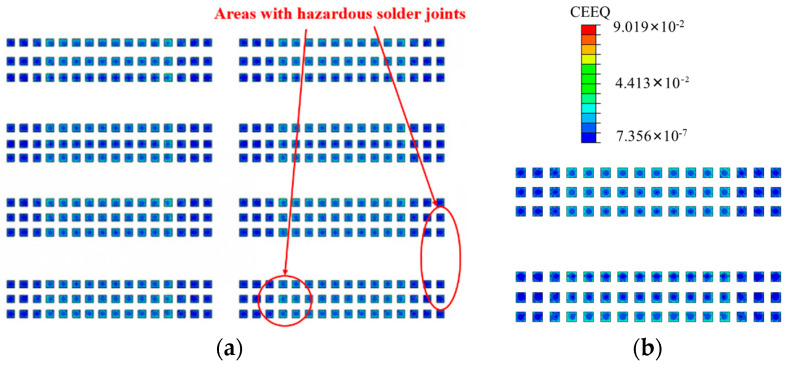
The CEEQ of the solder joints following the temperature cycling and load cycling stages: (**a**) A-side creep strain; (**b**) B-side creep strain.

Since the equivalent creep strain (CEEQ) and average viscoplastic strain energy density (CENER) of solder joints are related to the lifespan calculation method, the Anand constitutive [16,17] was used in this study to describe all the solder joints evident in this model, with the equivalent creep strain–stress nephogram presented in Figure 15. It can be observed that a significant creep strain occurs on both the upper and lower surfaces of the solder joints, with its overall distribution consistent with that of the stress. The large overall creep strain of the chip-end solder joints and the even greater creep strain of the edge solder joints are also evident. Unlike the stress distribution, the creep strain of a single solder joint shows a trend of a small creep strain in the center and a large creep strain in the epitaxial area, especially at the upper and lower joints. This trend was determined by the creep property of the solder joint and the different thermal expansion coefficients at the junction of the epitaxial and other compounds. Meanwhile, although there a large creep strain value was evident at the chip-end solder joints, the simulation results show that the maximum creep strain occurs at the edge of the package-end solder joints, with a maximum value of 0.0919. The nephogram distribution trend of the CENER is consistent with that of the CEEQ, with the maximum value evident at the same node.

In view of the abovementioned scenario, when the CEEQ and CENER are considered as criteria for hazardous solder joints, it is essential to consider the diagonal corner of the chip-end solder joints and the edge of the package-end solder joints.

#### 2.3.2. Analysis of Temperature Cycle Sub-Models

**Figure 16:**
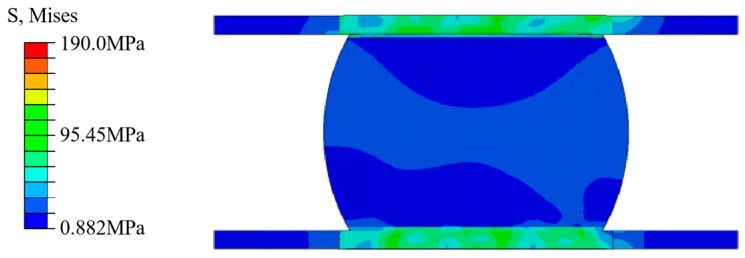
The stress nephogram profile of the temperature cycling sub-model.

**Figure 17:**
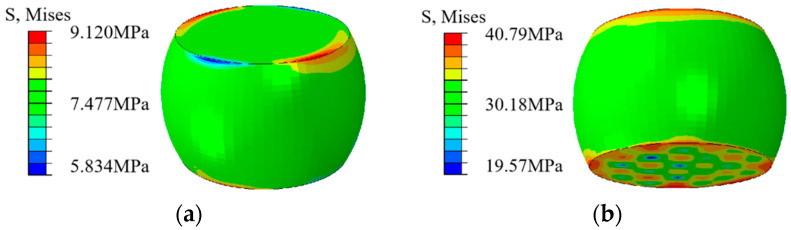
Stress nephograms of the temperature cycling sub-model: third cycle at (**a**) high and (**b**) low temperatures.

In order to further analyze the stress–strain situation of the solder joints, the solder joints with the maximum CEEQ value of the entire model were selected for a sub-model analysis, with the temperature cycling sub-model stress profile presented in Figure 16. It can be observed that the stress of the solder joints is mainly concentrated in the contact between the solder joints and the IMC coating, with less stress present inside the solder joints. The stress nephograms illustrating the solder joints at the highest (+150 °C) and lowest (−65 °C) temperatures are presented in Figure 17. The stress nephogram depicting the high temperature of the third cycle is presented in Figure 17a, indicating that the maximum stress occurs near the chip-end solder joints, and it is significantly concentrated at the top edge of the solder joints and evenly distributed. The stress nephogram presenting the low temperature of the third cycle is depicted Figure 17b, indicating that the highest stress values are mainly distributed at the edge of the bottom solder joints, without being concentrated in the middle area. Meanwhile, the higher stress values present at the top of the solder joints are also distributed along the outer edge. This stress distribution is mainly caused by the differences in the thermal expansion coefficient and elastic behavior of the different components. It can be observed from the stress values that the stress occurring at a low temperature is significantly higher than that at a high temperature. Therefore, whether in high- or low-temperature conditions, the maximum stress values appear at the edges of the upper and lower surfaces of the solder joints, and more stress can be produced at low temperatures.

**Figure 18:**
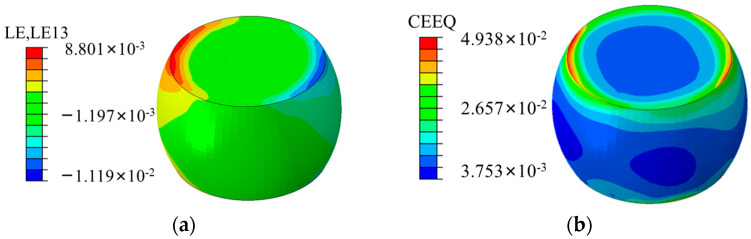
Stress nephograms of the temperature cycling sub-model: (**a**) maximum shear strain LE13 nephogram after cycling; (**b**) equivalent creep strain nephogram of the solder joints.

LE denotes the plastic shear strain, and LE1, LE2, and LE3 are denoted as the plastic shear strain in the x-direction, y-direction, and z-direction, respectively. The maximum shear strain LE13 nephograms produced following the temperature cycling stage are presented in Figure 18a. It can be observed that the shear stress is mainly concentrated at the upper surface edge of the solder joints, easily resulting in joint failure and fracture. The equivalent creep strain nephograms of the solder joints are presented in Figure 18b, indicating that the equivalent creep strain, also concentrated at the edge of the boundary joint, is consistent with the stress–strain distribution. According to the stress distributions of the entire model and the sub-model solder joints, the stress–strain behavior is concentrated at the boundary joint of the edge solder joints, which can be considered the most likely location for solder joint failure to occur.

#### 2.3.3. Data Analysis of the Temperature Cycling Model

**Figure 19:**
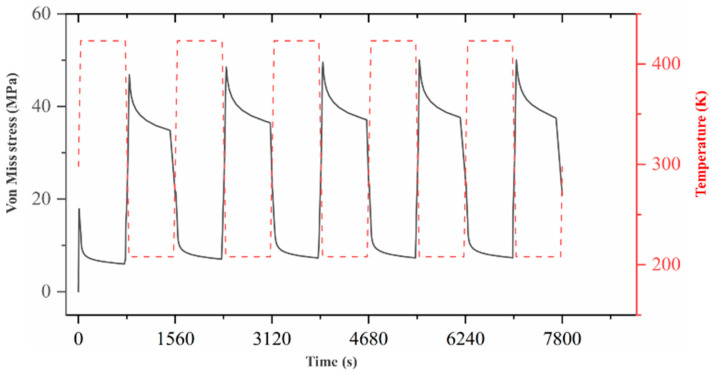
The temperature-dependent cycling load curves of the von Mises stresses (the maximum stress element) for the selected solder joint.

Figure 19 presents the temperature-dependent curves of the von Mises stresses (the maximum stress element) for the selected solder joint in the entire model with five temperature cycles. It can be observed in Figure 19 that the equivalent stress tends to stabilize after the third cycle.

**Figure 20:**
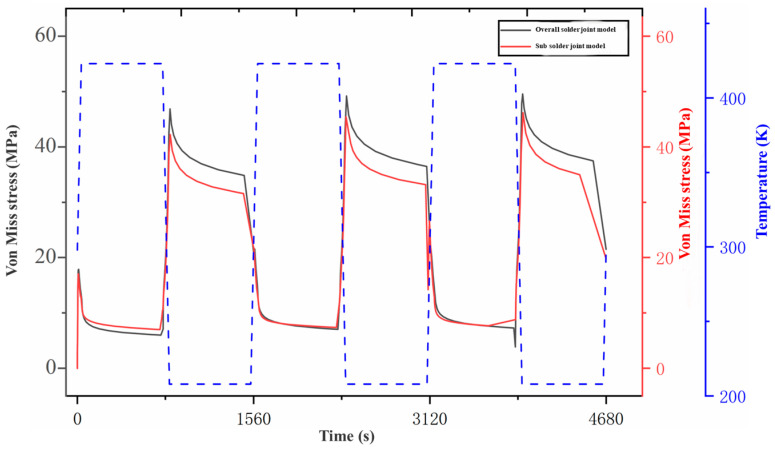
The temperature-dependent cycling load curves of the von Mises stresses (identical unit) for the selected solder joint.

Figure 20 presents the temperature-dependent curves of the von Mises stresses (the maximum stress element) for the selected solder joint in the overall model and the corresponding sub-model. The maximum stress values for this solder joint in the entire model and the sub-model were 49.546 MPa and 46.1607 MPa, respectively, with a difference below 10%. This difference was caused by the addition of copper pads and IMC coating to the sub-model, as well as the plastic properties of the copper pads. Therefore, it was feasible to use the sub-model to analyze the corresponding solder joints. This stress curve shows that the overall stress varies periodically and is related to the temperature load. At the beginning of the cycle, the stress accelerates with the increase in the temperature, due to the expansion of the compound and the release of stress caused by its high elasticity. When the temperature begins to decrease, the elastic material can shrink, so that the solder joint experiences a certain plastic deformation due to its viscoplasticity. Meanwhile, low elasticity at a low temperature results in an increase in the stress value while the temperature decreases. Moreover, when the temperature is maintained at a low value, a form of stress relaxation occurs, but the overall stress still remains high. Subsequently, the stress gradually decreases with the increase in the temperature. Throughout the entire cycle, the solder joint maintains a low stress value at the high-temperature stage and a high stress value at the low-temperature stage. Following the execution multiple cycles, the solder joint was prone to damage and crack initiation during the stage of maintaining a low temperature. The rapid crack propagation of the solder joint occurs easily under low-temperature conditions after performing multiple temperature cycling loads.

**Figure 21:**
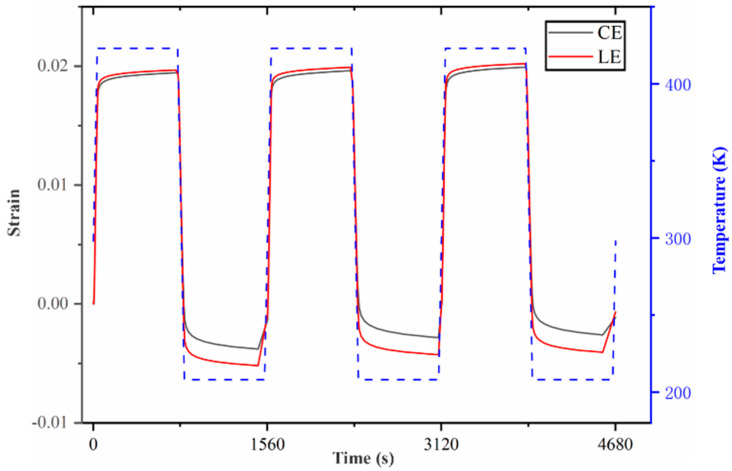
The time-dependent curves of the maximum shear strain element and creep shear strain.

The plastic shear strain (LE) and creep shear strain (CE) variation with time in the XY direction of the welded joint during cycling in Figure 21. It can be observed that, for the maximum shear strain element, the shear strain in the XY direction is positively correlated with the temperature value. The solder joint experiences its maximum expansion at a high temperature and its maximum contraction at a low temperature, with the creep being caused by the shear strain accounting for the majority of the stress. With the increase in the number of cycles being performed, the shear strain range remains basically unchanged, but the highest and lowest shear strain values gradually increase. It can be predicted that, with the continuous increase in the number of cycles, the shear strain value also continues to increase, and the solder joint generates significant shear strain activity under extreme temperature conditions, resulting in interface damage and, subsequently, solder joint damage.

**Figure 22:**
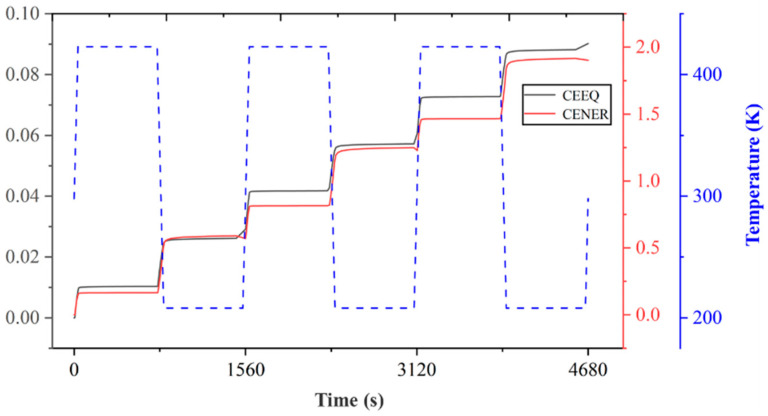
The time-dependent curves of the equivalent creep strain and viscoplastic strain energy density under the temperature cycling condition.

Creep deformation refers to one of the main causes of solder joint failure under a temperature cycling load. According to Figure 15, it can be observed that the larger values of the two factors are mainly concentrated at the edges of the chip-end and package-end chips, with the maximum value evident at the outermost edge of the solder joint. The time-dependent curves of the equivalent creep strain and viscoplastic strain energy density under the temperature cycling conditions are presented in Figure 22. Both the equivalent creep and viscoplastic strain can increase monotonically over time, with the largest increment occurring in the first cycle. During the temperature cycling stage, the equivalent creep can rapidly increase at variable temperatures, while it increases slowly during the insulation stage. With the continuous application of the cycling load, the equivalent creep strain increases, ultimately leading to excessive creep in larger areas and, subsequently, solder joint damage. Moreover, it can be determined that the variation trend of the viscoplastic strain energy density is consistent with that of the equivalent creep strain.

## 3. Fatigue Lifetime Prediction

### 3.1. Introduction to the Solder Joint Fatigue Lifetime Prediction

As crucial components for interconnecting various parts of 3D packaging storage devices, solder joints are not only used for electrical and mechanical connections but also provide thermal dissipation channels for chips. Hence, a device’s quality is largely determined by their reliability. When the solder joint is subjected to temperature cycling stress, strain accumulation occurs due to factors such as mismatched thermal expansion coefficients and the different elastic moduli of various structural compounds, resulting in the initial or new cracks in the solder joint continuously expanding until fractures occur, ultimately leading to device failure [18]. According to the different failure modes of solder joints, the prediction models for a solder joint’s lifespan are usually divided into several types, such as plastic deformation, creep deformation, fracture mechanics, and energy. These models can reflect the fatigue patterns of solder joints from different perspectives, including accuracy and application range, so that they are suitable for predicting lifespan fatigue values under different stress conditions or failure types. The Darveaux model [19], an energy-based model used to determine the fatigue lifetime of solder joints, was used in this study to perform a prediction analysis.

The Darveaux model mainly refers to an energy-based fatigue lifetime prediction model, in which four constants and two equations related to the crack propagation can be established by measuring the crack propagation rate of solder joints. In this method, it is necessary to consider the solder joint height and usage of the average value of stable changes in the plastic deformation energy per unit volume of eutectic solder. The specific equations are described as follows:

The equation for the thermal cycle, N_0_, causing an initial crack:

(1) $$N_{0}=K_{1}{\left(∆W_{ave}\right)}^{K_{2}}$$

The equation for the crack propagation rate, (da/dN), of a single thermal cycle:

(2) $$\frac{da}{dN}=K_{3}{\left(∆W_{ave}\right)}^{K_{4}}$$

In Equations (1) and (2):

$K_{1}$, $K_{2},\;K_{3},\;K_{4}$—the crack propagation constants obtained from the test relate to a single solder point and substrate thickness, with their specific variation values presented in Table 3.

$∆W_{ave}$ is the accumulation of average viscoplastic strain energy density of a single thermal cycling solder joint. Its equation is as follows:

(3) $$∆W_{ave}=\frac{\sum_{i=1}^{N}v_{i}∆w_{i}}{\sum_{i=1}^{N}v_{i}}$$

In Equation (3):

i—element number;

$v_{i}$—the volume of element i;

N—total number of selected elements.

The typical thermal fatigue lifetime value, (α), refers to the number of cycles at a 63.2% failure rate, with its equation presented as follows:

(4) $$\alpha =N_{0}+\frac{a}{da/dN}$$

In Equation (4):

α—the length of the fracture feature (usually the diameter of the solder joint connection).

### 3.2. Introduction to the Solder Joint Fatigue Lifetime Prediction

According to the 1010 testing condition C of the MIL-STD-883 method, the load conditions were simulated with a temperature range of −65 °C~+150 °C, a switching time of 1 min, and a residence time of 12 min. To thoroughly ensure the validity of the predicted values, all the relevant parameters were obtained from the maximum values of the corresponding parameters during the simulation process, and predictions were made for the A- and B-sides of the microelectronic device. The data presented in Table 4 can be obtained from the simulation results.

**Figure 23:**
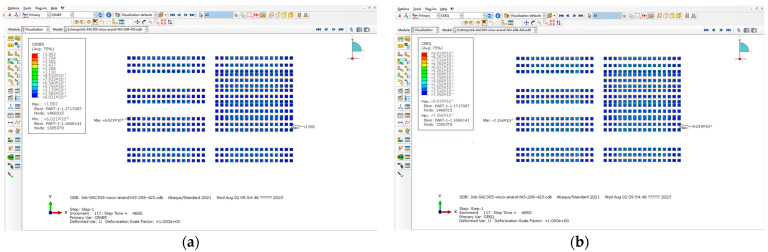
Temperature cycle: (**a**) typical process for selecting maximum CENER; (**b**) the typical process of selecting the maximum CEEQ.

**Figure 24:**
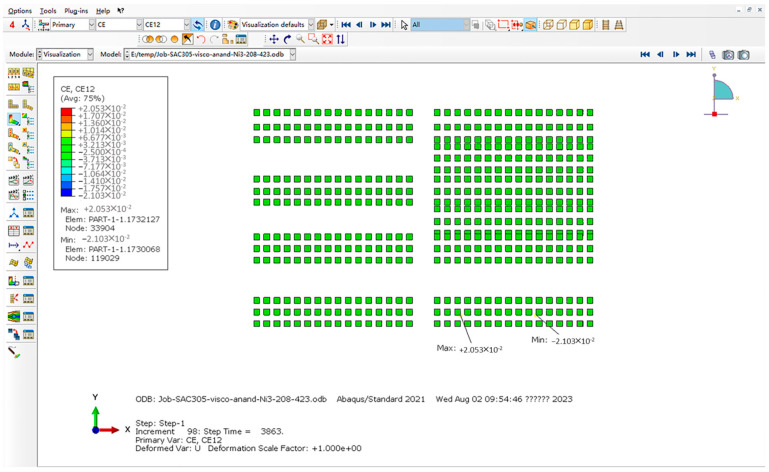
Maximum CE12 selection process (XY direction shear strain).

For Equations (1) and (2), the K value represents the stress intensity factor at the crack tip, which is an important parameter for measuring the crack propagation ability. The magnitude of K value depends on the stress field at the crack tip and the elastic properties of the material. According to the model data, the minimum unit is approximately 0.0445 mm, which is approximately 1.75 min when converted to milli-inches. The number 1.5 in Table 3 is similar, so these data are used. For Equation (3), the ∆Wave value first selects the maximum solder joint with the highest energy density of viscoplastic strain. The typical process is shown in Figure 23, and then extracts the maximum unit data. The typical process is shown in Figure 24 (the average difference within a single cycle is approximately the final value divided by 3). For Equation (4), the value of a is the length of the fracture characteristic and the length of the solder joint connection, so the value is taken as 0.45 mm. In addition, in the Darvieux model, the crack propagation correlation coefficient provided by Darvieux is in English units, so it is necessary to divide ∆Wave obtained from ABAQUS by 6.894757 × 10^−3^ to convert the unit from MPa to Psi, and also divide the solder joint diameter by 25.4 to convert it from millimeters to inches.

According to the data in the table above, it can be concluded that the A-side solder joints fail earlier than the B-side solder joints, which is caused by the asymmetric structure of microelectronic devices. Therefore, the A-side solder joints are used as the failure criterion.

## 4. Results and Discussion

According to the 1010 testing condition C of the MIL-STD-883 method, 100 cycles were initially performed, and then they were stopped. After completing each specified temperature cycle, a functional test was performed. If the functional performance of the device failed, the test was stopped. Finally, a scanning acoustic microscope examination and cross-section test were conducted on the failed device for further analyses.

### 4.1. Analysis of the Test Results

#### 4.1.1. Temperature Cycling Test Results

**Figure 25:**
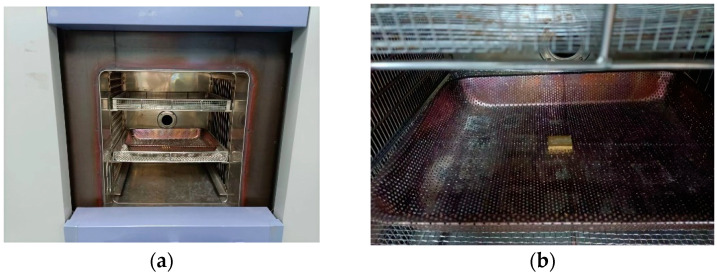
Temperature cycling ultimate test: (**a**) testing process; (**b**) sample arrangement.

**Figure 26:**
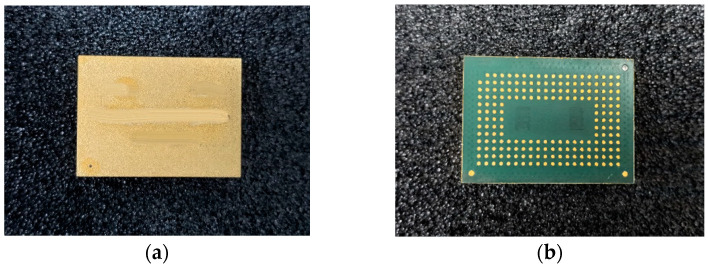
The sample’s appearance: (**a**) front; (**b**) back.

The functional performance test of the device failed after 1500 temperature cycles. Comparing the simulation prediction simulation results with the actual temperature cycling test results, it can be seen that the deviation is within 10%, and the specific data comparison is shown in Table 5, with the typical appearance of the testing process presented in Figure 25. In addition, there was no obvious abnormality evident in the device’s appearance, with its typical appearance presented in Figure 26.

#### 4.1.2. Examination Results Using a Scanning Acoustic Microscope

**Figure 27:**
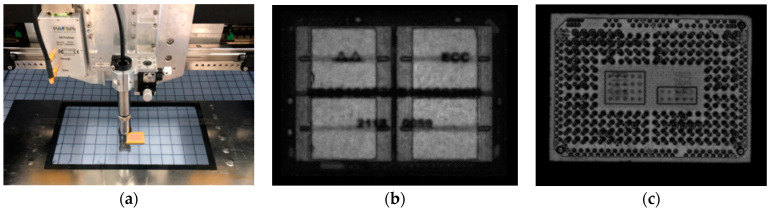
Scanning acoustic microscope examination: (**a**) testing process; (**b**) top-view scanning; (**c**) rear-view scanning.

**Figure 28:**
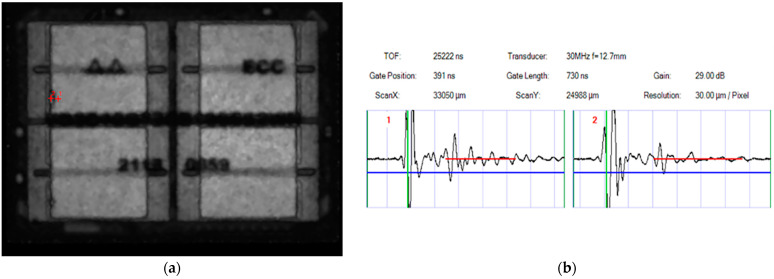
Examination results using the scanning acoustic microscope: (**a**) delamination between the chip and molding compounds (red mark labeled “2”); (**b**) comparison of delaminated and non-delaminated waveforms.

The device was examined using a scanning acoustic microscope according to MIL-STD-1580, with the typical testing process presented in Figure 27. After the examination, it was observed that a storage chip located inside the device and the molding compounds were obviously delaminated (top view), and that the delamination failure position was consistent with the simulation results, with its typical appearance presented in Figure 28.

#### 4.1.3. Test Results of the Functional Performance

**Figure 29:**
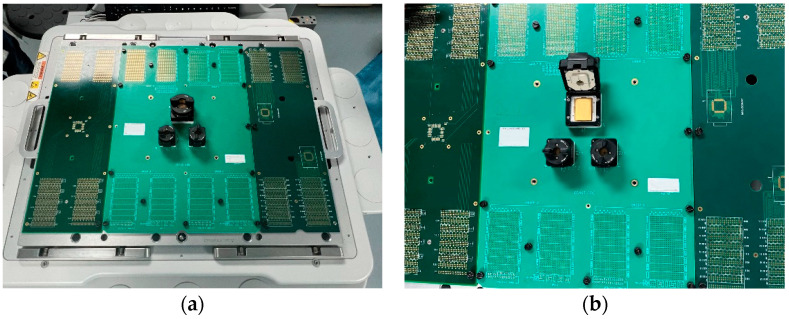
Functional performance test: (**a**) ATE 93000 test board; (**b**) sample installed on the test stand.

The device was placed on the ATE93000 machine through a test board to perform the functional testing step, with the process presented in Figure 29.

After the temperature cycling test was conducted, it was observed that the product’s lead out terminals, DQ2 and DQ9, were open-circuited. The testing results are presented in Table 6.

#### 4.1.4. Results of the Cross-Section Analysis

**Figure 30:**
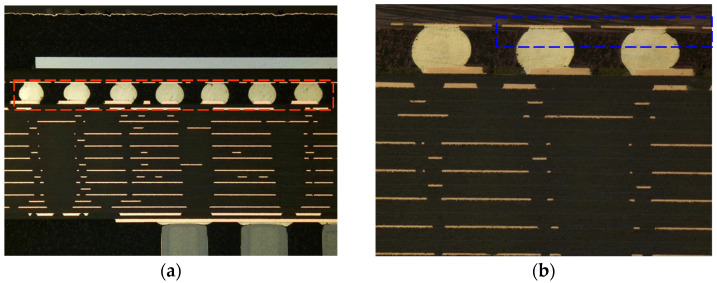
Cross-section appearance: (**a**) the BGA and the substrate are significantly tilted (red frame); (**b**) cracks between the solder balls and substrate solder (blue frame).

After the cross-section observation of the failed module was performed, it was observed that the internal interconnection structure of the module was affected by shear fatigue stress. Thus, the ball grid array (BGA) and the substrate were severely tilted, with cracks appearing between the solder balls and the substrate solder, while no obvious abnormality was observed in the internal storage chip of the module. The device’s typical appearance is presented in Figure 30.

It can be observed in Figure 30 that the failure appearance and area of the solder joints after the temperature cycling test was performed are consistent with the simulation results. The solder position has shifted in the interconnected area between the chip and PCB, resulting in significant stress being placed on the contact surface of the solder joints, with numerous cracks appearing close to the chip-end solder joints. Moreover, the greater the strain is closer to the chip’s edge, the greater the solder joint failures.

**Figure 31:**
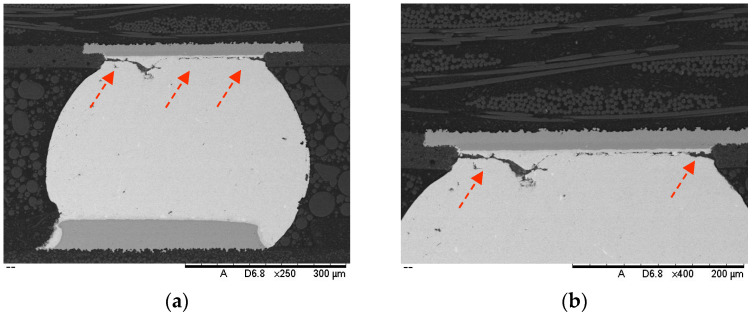
Scanning electron microscopy observation: (**a**) cracks penetrate the entire solder ball; (**b**) solder joint cracks grow along the IMC layer.

Scanning electron microscopy was performed for the further observation and analysis of failed solder joint cracks. It can be observed in Figure 31 that the cracks initiate from the intermetallic compound (IMC) at the edge of the solder joint and gradually extend towards the interior of the solder joint. In general, the internal structure of the SAC305 solder ball is characterized by a β-Sn + Cu6Sn5 + Ag3Sn ternary eutectic structure with a uniform distribution of grain size, with directional dendrites located at the edge of the solder ball [20,21]. However, with the periodic increase in the temperature cycling activity, recrystallization and grain growth preferentially occurred at the top of the SAC305 solder ball, thereby providing a favorable path for crack propagation. Under the joint action of dendrites and the temperature, cracks gradually shifted from the edge to the top of the solder ball. Moreover, the dendrites in the entire solder joint become thicker and longer in the direction of the temperature gradient, resulting in excessive local stress. Finally, cracks penetrated the entire grain, and ductile fractures shifted to brittle fractures. In addition, different coefficients of the thermal expansion (CTEs) of compounds, such as solder joints, chips, PCB, and epoxy resins [22], resulted in the formation of a certain stress gradient inside the solder joints, as well as atomic diffusion and lattice slip creep. Therefore, cracks are more likely to occur on the contact surface under the thermal creep stress condition.

Through the analysis of the above test results, it can be seen that to carry out this kind of complex 3D package storage module limit capacity research and analysis, not only is a temperature cycling box required but also an acoustic scanning microscope, a scanning electron microscope, a metallurgical microscope, and a 93000 ATE automatic test system. In this paper, the development of test procedures and test boards for 3D encapsulated memory modules has significantly improved the accuracy compared to the daisy-chain circuitry and contact resistance strain gauges used by universities or non-device manufacturing companies to monitor whether a product fails [23].

A combination of experimental and finite element simulation was used, which is the current mainstream research method in industry. Simulation modeling can be used to model the stress distribution and weak reliability regions of 3D packaging storage modules, providing a theoretical basis for the precise locations of 3D packaging storage module failure analysis. Using the empirical formula of solder joint life prediction can estimate the test failure time of 3D packaging storage module for thermal shock and provide data support for the timing end of the actual test, which is basically the same with other researchers and scholars using the empirical formula of solder joint lifetime prediction to estimate the test failure time of temperature cycling [24,25,26], and from the actual test results, it can be seen that only the number of cycles under these test conditions should be increased, not the temperature range, conversion time, residence time, or other test conditions, as the failure mode and mechanism of the solder joints will essentially not change. In addition, the appraisal mechanism is consistent with the identification of the test, which beneficial as conventional testing methods cannot assess products in certain extreme application scenarios of short boards.

At present, most researchers and scholars concerned with the reliability of 3D packaging memory are mainly focused on the two structures of package stacking and chip stacking; however, this paper adopts the cube package structure, which, due to expensive and long manufacturing cycles and restrictions, has been researched very little [27]. In addition, the magnitude of the applied test load conditions is extremely insufficient. Since 3D packaging memory is generally used in civilian applications such as mobile phones, smartwatches, mobile hard drives, etc., most researchers and scholars select test conditions mainly based on industry or civilian standards, such as JEDEC, IEC, etc., rather than military standards, such as MIL, GJB, etc., where the test conditions are more demanding. However, if product in the development stage and in the manufacturing stage are tested in accordance with JEDEC, IEC, and other relevant standards, for quality verification, the device has a certain degree of reliability if it remains under same test stress conditions of the reliability study. However, as this not sufficient for the reliability of the product when utilized in more extreme environments where high reliability is required [28,29,30], this paper combines the typical operating temperature range of current high-reliability integrated circuits (−55 °C~+125 °C) and the harsh test conditions of the U.S. military standard temperature cycling (−65 °C~+150 °C, conversion time of 1 min, and residence time of 12 min for each) to carry out a reliability study on the temperature cycling limit capacity of 3D stereoscopic packaging structure memory, which is of great significance in promoting the 3D packaging of memory in the field of reliability research.

## 5. Conclusions

In this study, the operating and destruction limits of a 3D packaging storage module were successfully detected under temperature cycling stress conditions. Moreover, the reliability of the storage module under temperature the cycling ultimate stress condition was evaluated using various tests and a finite element analysis. In the ultimate test of temperature cycling, cracks were considered the main factor causing the fatigue failure of solder joints in the module and delamination failure at the internal interfaces of the studied device. Based on the finite element analysis, a 3D packaging storage module was simulated to study and analyze its stress–strain properties. The solder joints presenting the maximum equivalent creep strain in the overall model were selected for a detailed sub-model analysis. The simulation results indicate that 3D packaging memory devices can suffer from thermal fatigue failure in the case of abrupt temperature changes. Cracks are caused by the accumulation of plastic and creep strains, and the CTE difference between epoxy resin and solder joints accelerates the generation of cracks. The finite element simulation results are consistent with the test results, presenting a deviation below 10%, according to the mutual verification of the electrical performance testing method, scanning acoustic microscope examination, cross-section examination, and scanning electron microscope observation following the execution of the temperature cycling test. This device was able to withstand 1500 cycles under the temperature cycling conditions (a temperature range of −65 °C~+150 °C, a switching time of 1 min, and a residence time of 12 min), far exceeding the 15 cycles specified in the qualification test. Therefore, it was proved that the device was highly reliable.

## Figures and Tables

**Table 1:** Internal and external structure dimensions of the storage module.

Category	Components	Dimensions
Solder ball	Diameter	0.45 mm
	Spacing	0.80 mm
	Number of single rows	16 each
	Number of rows for a single substrate	3 rows
	Total number of rows for a single chip	6 rows
	Height	0.3 mm
PCB (1)	Quantity	10 each
	Length × Width × Height	13 × 4.15 × 0.20 mm
Die	Quantity	5 each
	Length × Width × Height	8.50 × 8.00 × 0.20 mm
Storage	Length × Width × Height	13.00 × 9.00 × 0.50 mm
	Quantity	5 each
PCB (2)	Length × Width × Height	29.00 × 20.50 × 2.00 mm
Storage module	Length × Width × Height	30.00 × 22.00 × 0.70 mm

**Table 2:** The properties of the various compounds.

Physical Indicators	Compound Category and Numerical Value					
	PCB	Plastic Package Layer	Resin Sealing Layer	Chip	Substrate	SAC305 Solder
Elastic modulus	27 GPa	16 GPa	16 GPa	131 GPa	22 GPa	39.5 GPa
Poisson’s ratio	0.18	0.25	0.25	0.30	0.28	0.3
Coefficient of thermal expansion (CTE)	X: 10 × 10^−6^·K^−1^ Y: 10 × 10^−6^·K^−1^ Z: 23 × 10^−6^·K^−1^	15 × 10^−6^·K^−1^	21 × 10^−6^·K^−1^	2.7 × 10^−6^·K^−1^	16 × 10^−6^·K^−1^	28 × 10^−6^·K^−1^

**Table 3:** Darveaux fatigue lifetime model parameters.

Analyzing Unit Size	K_1_	K_2_	K_3_	K_4_
0.5	71,000	−1.62	2.76	1.05
1.0	56,300	−1.62	3.34	1.04
1.5	48,300	−1.64	3.80	1.04

**Table 4:** Prediction results of the solder joint lifespan.

Model	$∆W_{ave}$	Cycle Index
A-side	0.21733	1637
B-side	0.20833	1821

**Table 5:** Comparison of simulation data with experimental data.

Loading Conditions	Simulation Predicts the Number of Cyclic Failures	Number of Actual Test Failure Cycles
Temperature range of −65 °C~+150 °C, switching time of 1 min, and residence time of 12 min	1637 cycles	1500 cycles

**Table 6:** Electrical performance test results.

Lead-Out Arrangement	Technical Parameters	Testing Conditions (V_DD_ = 1.575 V and 1.283 V, V_REF_ = 0.50 × V_DD_, V_IH_ = V_REF_ + 0.16 V, V_IL_ = V_REF_ − 0.16 V)	Minimum	Maximum	Unit	Allowed Deviation
Pin	passVolt	/	200	900	mV	±0.5
DQ2	passVolt	/	2077.047		mV	/
DQ9	passVolt		8334.295		mV	

## Data Availability

The original contributions presented in the study are included in the article, further inquiries can be directed to the corresponding author.
